# Harnessing the Activity of Lytic Bacteriophages to Foster the Sustainable Development Goals and the “One Health” Strategy

**DOI:** 10.3390/v17040549

**Published:** 2025-04-09

**Authors:** Belén Álvarez, Elena G. Biosca

**Affiliations:** 1 Departamento de Microbiología y Ecología, Universitat de València (UV), 46100 Valencia, Spain; 2 Área de Investigación Aplicada y Extensión Agraria, Instituto Madrileño de Investigación y Desarrollo Rural, Agrario y Alimentario (IMIDRA), 28805 Madrid, Spain; mariabelen.alvarez@madrid.org

**Keywords:** virus, phage, biological control, phage therapy, biotechnological application, resistant bacteria, agrochemical, antibiotic, global health, SDGs

## Abstract

As bacteriophages (phages) are viruses that infect and destroy bacterial cells, they can be considered natural bactericides that can either directly or indirectly contribute to the achievement of the United Nations Sustainable Development Goals (UN SDGs) on health and well-being, food production and food security, as well as environmental protection and climate change mitigation, thus contributing to the success of the European “One Health” strategy to combat antimicrobial resistance in humans, animals, plants, and the environment. The biological activity of lytic bacteriophages can operate in the fields of microbiology and biotechnology for clinical, veterinary, agricultural, and industrial applications, among others, to achieve the proposed goals, mainly because the phages can help increase crop productivity by reducing bacterial diseases; constitute alternative therapies against infections caused by multidrug-resistant bacteria; can reduce populations of pathogenic bacteria that contaminate soil and water, therefore ensuring healthier and safer food production; and they can help reduce environmental pollution caused by the presence of agrochemicals and antibiotics. Phage-based therapies developed through research and innovation have the potential to promote greater global food security and health in a more environmentally friendly and eco-sustainable way.

## 1. Introduction

To achieve a more sustainable future, it is essential to make optimal use of all natural resources, including the microorganisms found in ecosystems, such as bacteriophages, the viruses of bacteria [1,2], abbreviated as phages. These viruses are highly abundant and possess valuable biological properties. Phages specifically target and replicate within bacteria while leaving other living organisms unharmed. They were first discovered in 1915 by Frederik William Twort and in 1917 by Felix d’Herelle, who noticed their potential to kill bacteria [1]. Since then, phages have been extensively studied, mainly due to their ability to treat bacterial infections, which can lead to numerous potential biotechnological applications [3,4]. They are recognized as potent natural antimicrobials, making them suitable for the sustainable and safe treatment of bacterial infections in humans, animals and plants. In fact, phages can serve as an alternative or complement to traditional antibiotics and other chemical antimicrobials [4,5,6,7]. This approach aligns with the Sustainable Development Goals (SDGs) established by the United Nations (UN) as part of the 2030 Agenda for Sustainable Development [8]. These goals represent a global effort to address social, economic, and environmental challenges. Additionally, they correspond with the European “One Health” Action Plan, which aims to combat antimicrobial resistance in humans, animals (2017/2254/INI) and, more recently, in plants of agronomic interest [9].

Based on their life cycles, bacteriophages can be classified into two main types: lytic and lysogenic [10,11]. Lytic, or virulent, phages act as natural antimicrobials with a bactericidal effect. After infecting their target bacterium, they utilize their metabolic machinery to replicate themselves and ultimately destroy the host cell through lysis when numerous new virions are released. On the other hand, temperate or lysogenic phages integrate their genome into that of the host bacterium and replicate as a prophage without causing cell lysis. Therefore, lytic phages are considered the most important for controlling or treating pathogenic bacteria [7,12].

Bacteriophages and their lytic proteins are primarily used to treat multi-resistant bacterial infections [4,13,14]. They also have other notable applications; for instance, bacteriophages and their enzymes can effectively disrupt the biofilms formed by their bacterial hosts [14,15]. Biofilms pose a significant challenge in eradicating bacterial infections, even when the bacteria are susceptible to antibiotics. This can lead to the re-establishment of biofilms after treatment. Some bacteriophages produce depolymerases that can either prevent biofilm formation or break down existing biofilms, thereby enhancing their ability to lyse the target bacteria [16,17,18]. Additionally, bacteriophages can be employed in the biodetection of pathogens [19]. This approach consists of identifying pathogens by detecting their specific phages, and other applications are related to the synthesis of their lytic proteins [20]. Phage-based biodetection is considered a very useful tool for monitoring and ensuring the safety of various products, such as food and drugs, and identifying pathogens in environmental samples [19,21]. Moreover, bacteriophages can be used for the improvement and/or modification of the gut microbiota. Thus, the consumption of phages as probiotics can contribute to the reduction in the use of antibiotics in humans and animals, as well as other types of pollutants in the environment [22,23,24,25]. In relation to that, bacteriophages have been proposed to decrease livestock-produced methane. Since ruminal fermentation usually leads to methane generation, phage treatment would cause perturbations of the microbial populations taking part in this process, resulting in decreased production [26]. Other uses of bacteriophages include biogas production, which is a renewable energy source, since phage cocktails have been shown to act as efficient enhancers of biogas production in the anaerobic digestion processes taking place in industrial bioreactors [27]. In a similar way, phages have a beneficial role in the inhibition of contaminating bacteria during industrial fermentation processes, which traditionally relied on chemicals and/or antibiotics. Such is the case of fermentations by yeasts, where phages can improve ethanol production by suppressing unwanted lactic and acetic acids through the inhibition of the target undesirable bacteria [28].

One significant advantage of bacteriophages is their high specificity [12,20,29]. They usually infect only one pathogenic bacterial species without harming the beneficial microbiome of the host or the surrounding environment, making them environmentally friendly. Bacteriophages replicate solely within the target bacteria and do not infect animal or plant cells, ensuring their safe use. Therefore, their utilization as antibacterial agents does not cause an ecological impact, being much less detrimental to bacterial communities compared to antibiotics [30]. In general, they are natural biological components of the ecosystems, contributing to the maintenance of the bacterial population balance [31]. Phages can also be involved in horizontal gene transfer events between bacteria, which can generate the acquisition of genes related to antibiotic resistance or virulence factors [32]. For this reason, prior to the selection of the phages for therapy, it is usually advisable to perform a suitable genomic analysis that can rule out the presence of such genes. As bacterial predators, lytic phages are typically needed in low concentrations, since their natural replication in the infected area increases the likelihood of contacting the target bacteria, and therefore very few doses are required after the initial administration [6,33]. Bacteriophages can be combined with other prevention or control strategies, both sustainable and chemical, which adds to their versatility [34,35,36,37]. They can be administered through various routes, depending on how the pathogenic bacteria enter the host, whether orally, intravenously, subcutaneously or transcutaneously in humans and animals, or via irrigation, soil drenching or the spraying of aerial parts in plants [33,38]. Significantly, they do not alter the organoleptic properties of foods and are generally unaffected by standard preservation methods [39]. In addition, their production costs are generally low, although large-scale phage manufacturing either for phage therapy or the biocontrol of pathogenic bacteria is frequently a specific and strongly controlled process, which makes it advisable to optimize previous key operational parameters in order to increase the cost-effectiveness rate [33,40]. Issues such as the cost of updating phage-cocktails or combining them with other antimicrobials should be addressed. In some cases, such as phage therapy for human health, there might be an increment in costs due to a lack of large-scale clinical trials, which might be compensated by a shorter treatment period compared to that required by antibiotics and, consequently, reduced healthcare costs. Thus, moving from a laboratory-scale validation and a successful scale-up of phages in bioreactors to an industrial level would benefit from optimized scalable methods or simulation models aimed at minimizing costs while maximizing efficiency [40,41].

The use of bacteriophages, while promising, may be limited due to their narrow host range [12]. This challenge can be addressed by employing phage cocktails, consisting of formulations targeting multiple bacterial strains simultaneously, which, additionally, help to overcome development of bacterial resistance [20,33,42]. Several mechanisms by which bacteria can become resistant to phage infection are known, which could inhibit successful treatment outcomes. These include mutation of the surface receptors involved in phage recognition, interference in phage replication and bacterial suicide [43,44]. However, some cases of phage therapy applied to animals indicate that these bacterial mutations can result in fitness costs in the phage-resistant variants, which could eventually benefit the host [43]. Additionally, it has been reported that bacterial cells experience altered susceptibility to antibiotics after developing phage resistance [45]. Therefore, the use of phage cocktails or their combination with other substances stands out as an appropriate strategy to combat phage bacterial resistance [43,45]. Clinical studies have generally demonstrated that bacteriophages are safe and tolerable for human and animal use [46], presenting a lower risk of adverse side effects compared to certain antibiotics. Although there is some evidence of mild immune responses to phages, these responses are usually manageable [47]. Given the considerable potential that phage therapy demonstrates beyond human and animal health, there is a need to establish a thorough international regulatory framework. The legal handling of this therapy poses questions such as whether fixed-ingredient phage cocktails belong to a particular type of medicinal bioproducts and how to regulate the technical ways in which phages can be manufactured and administered [48]. Only by recognizing the importance of conducting more well-controlled clinical trials, leading to the official licensure of phage therapy, and by forming international expert committees where specialists can reach agreements, will countries across the world be able to best overcome the current regulatory hurdles [48,49].

With increasing regulatory pressure on governments and organizations to meet the Sustainable Development Goals of the 2030 Agenda, identifying a common framework to address them has become an urgent priority [50]. Many countries have adopted the “One Health” strategy and the SDGs as part of their national policies, including those in the European Union Green Pact. The overarching objective is transitioning to a greener, healthier, and more sustainable economy. However, the rise in antimicrobial resistance among bacterial pathogens seriously threatens global health and hinders efforts to achieve the SDGs [51]. Antimicrobial resistance is one of the main global threats to health and has significant implications for the world economy [33,51,52]. In response to this growing concern, there is an increasing demand for novel, innovative, and effective antimicrobial treatments that are able to reduce or eliminate the bacterial infections and/or limit the spread of emerging diseases under the “One Health” framework [52]. In recent years, research on bacteriophages has significantly increased, especially focusing on designing optimal phage cocktails to combat bacterial infections successfully. The goal is to develop new therapies based on phage activity that are more sustainable and environmentally friendly for controlling bacterial infections in humans, animals, and plants, as well as for managing pathogens and/or undesirable bacteria in food and the environment [5,6]. Some of these therapies have already been patented [53,54,55,56], and the commercialization of phage-based bioproducts is progressively increasing in the fields of microbiology and biotechnology [5,39,52].

## 2. Bacteriophages and Sustainable Development Goals

**Figure 1:**
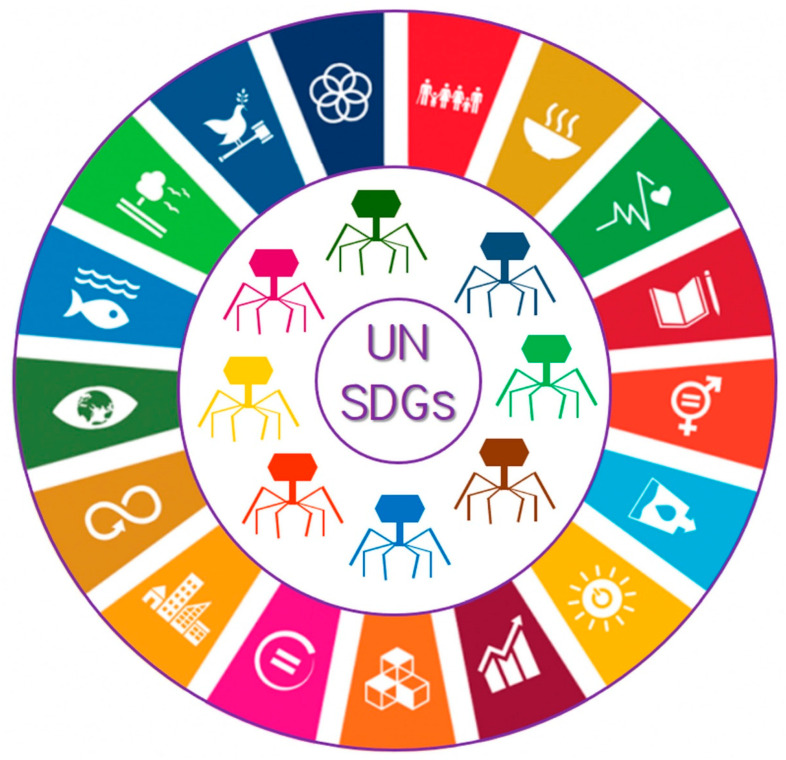
Bacteriophages and the United Nations (UN) Sustainable Development Goals (SDGs) globally proposed in the 2030 Agenda for Sustainable Development. Adapted from [8]. See text for details.

In terms of achieving the 17 internationally agreed UN SDGs [8], bacteriophages can contribute to these goals, either directly or indirectly, as follows (Figure 1):

With respect to UN SDG 1 “No Poverty”, which aims to end global poverty and privation, phages can be relevant through their applications in health, sanitation, food production, and environmental sustainability [50,57,58]. Phage therapy offers a more affordable alternative to antibiotics for treating multidrug-resistant bacterial infections, not only in humans but also in crops and livestock, reducing losses from bacterial infections, improving agricultural yields, and securing the livelihoods of rural households. In addition, in food safety, by improving food preservation and controlling food-borne diseases [5,59], as well as in the bioremediation of bacterial contaminated environments [60], promoting health, food safety, and a healthier environment in areas with fewer economic resources.

In relation to UN SDG 2 “Zero Hunger”, bacteriophages can be employed for the biological control of pathogens in food, helping to reduce food contamination and the incidence of foodborne bacterial infections, thereby enhancing food security [5,6,20,61,62]. Phages serve as effective and specific natural predators of various bacterial species. They can be utilized as therapeutic agents in aquaculture, crop farming, livestock farming, and agriculture, and as biopreservatives in food production [5,42,59,62,63,64]. The use of phages reduces the reliance on antibiotics in animal and crop production, thus promoting more sustainable and safe food production while decreasing the application of chemical antimicrobials [4].

UN SDG 2 “Zero Hunger” is closely related to UN SDG 3 “Global Health and Well-Being”. The implementation of bacteriophage-based therapies is considered a promising alternative or complement to antibiotics, especially given the current rise in antimicrobial resistance among significant pathogenic bacteria affecting humans, animals, and plants [5,6]. Phages specifically target their host bacteria, eliminating pathogenic strains without harming beneficial microbiota. Recent literature includes numerous successful examples of phage therapy managing chronic and complex infections in humans, animals, and plants [5,6,7,42,65]. Additionally, phage therapy allows for the reduction in the usage of antibiotics and chemicals in medicine, veterinary practices, and agriculture, while keeping under control the spread of multidrug-resistant bacteria, which is crucial for global health [51].

On the other hand, bacteriophages can have a role in the UN SDG 4 “Quality Education”, which aims for inclusive and equitable training for sustainable development. Both, education and the dissemination of information are essential to promote social acceptance for the widespread use of phages, and therefore favorable reception among consumers [66,67]. Raising public awareness about the benefits of bacteriophages and the necessity for regulatory changes to combat multidrug resistance is a significant challenge. There is a pressing need to inform the public about the safety and advantages of phage therapy and other biotechnological applications to foster understanding and acceptance [5,66,68]. Science projects that directly involve citizens can play a vital role in this effort by promoting scientific literacy and community engagement, which can accelerate the development of new therapies for treating resistant bacterial infections in humans, animals, and plants [69,70]. Educational initiatives to collaborate with society to raise awareness about phages as beneficial viruses that are potentially effective against multidrug-resistant bacteria can contribute to inspiring pre-university students to pursue careers in science [68,71,72], as well as to raising awareness among citizens about antimicrobial resistance.

UN SDG 4 “Quality Education” can be related to UN SDG 5 “Gender Equality”. Although the relationship between phages and UN SDG 5, which aims to achieve parity and equal opportunities for women, may not seem obvious, connections can be made when placed in a broader societal context, such as the scientific research in bacteriophages and their multiple applications, an emerging field in microbiology and biotechnology [4,61,73]. Supporting and encouraging the participation of women in phage research and STEM (Science, Technology, Engineering, and Mathematics) careers can help to promote gender equality and the inclusion of women into the professional world related to these scientific matters [74].

UN SDG 2 “Zero Hunger” and UN SDG 3 “Global Health and Well-Being” are closely related to UN SDG 6 “Clean Water and Sanitation”. Bacteriophages can enhance the availability of clean water by playing a role in the remediation of water contaminated with pathogenic bacteria. They can be used to treat urban sewage and other wastewater effectively by reducing or eliminating bacterial pathogens [60], as well as agricultural irrigation water contaminated with plant pathogenic bacteria [6,42]. This application improves water quality, decreases the risk of transmission of waterborne disease to humans, animals, and plants, mitigating the spread of bacterial resistance and revealing that phages are valuable for addressing current health and environmental challenges.

Bacteriophages can contribute to UN SDG 7 “Affordable and Clean Energy” at least indirectly, by improving the efficiency and sustainability of waste-to-energy technologies for biofuel production [27,53]. Phages can help manage bacterial infections that can disrupt fermentation processes, decreasing energy waste and enhancing biofuel and bioethanol outcomes [28]. This supports the transition to cleaner and more sustainable energy generation, reducing dependence on fossil fuels.

UN SDG 4 “Quality Education” and UN SDG 5 “Gender Equality” are, in most cases, interconnected with UN SDG 8, “Decent Work and Economic Growth”. Bacteriophages and their applications can assist in achieving UN SDG 8 through the creation of new jobs in the fields of microbiology and biotechnology and/or the implementation of practices favoring the development of sustainable agriculture, livestock, and fish farming while reducing production costs [5,38,75]. The biotechnological applications of phages contribute to opening new lines of work to implement innovative safe strategies for an environmentally sustainable economic development [3,4,13,20].

The previous UN SDGs are also linked to UN SDG 9 “Industry, Innovation and Infrastructure”, which emphasizes building resilient infrastructure, promoting sustainable industrialization, and fostering innovation. Phages, either alone or in combination with other biological or chemical strategies, can stimulate research and innovation, leading to new biotechnological applications in the agri-food industry, environmental bioremediation, and global health [20,24,28,57,61].

The use of bacteriophages can be relevant to UN SDG 10 “Reduced Inequalities”, which focuses on addressing social disparities, mitigating differences and bridging gaps among and within countries, being related to other UN SDGs. Phage applications can reduce inequalities in health, facilitate access to clean water and sanitation, and contribute to food security and the production of organic and sustainable food [21,42,62]. They can contribute to the development of more accessible antimicrobial therapies against multi-resistant bacterial infections in humans, animals, and agriculturally relevant plants and improve food security for populations in countries with less developed health systems, food safety services, and food production [76].

Phages have the potential to contribute to UN SDG 11 “Sustainable Cities and Communities” by promoting public health, food security, and environmental sustainability in the urban environment [52]. Their use supports urban resilience, manages bacterial infections, reduces urban pollution, and promotes sustainable urban development.

The contribution of bacteriophages to the achievement of the UN SDGs includes UN SDG 12 “Responsible Consumption and Production”. Thus, phages represent a sustainable alternative to antibiotics in aquaculture, agriculture, and animal husbandry, which can help reduce bacterial multiresistance and the environmental impact associated with the use of antibiotics and agrochemicals [4,5,6,58].

Moreover, all of these UN SDGs relate to UN SDG 13, which is centered on “Climate Action”. Using phages in bioremediation can sustainably reduce bacterial contamination in natural environments such as water and soil, particularly from sanitary and agro-food wastewater. This contributes to the remediation of these contaminated environments while decreasing reliance on agrochemicals and antibiotics [6,51,58,60,77]. Furthermore, the implementation of phage activity in agriculture can mitigate the adverse effects of climate change, promote sustainability, and ensure food security in an evolving climate [58].

Phages also benefit UN SDGs 14 and 15, which focus on “Life below Water” and “Life on Land,” respectively, due to their multiple applications in animal and plant production [6,24,26,61,62,75,78].

In the same way, bacteriophages can contribute to UN SDG 16 “Peace, Justice and strong Institutions” by providing more affordable, economical, safe, and environmentally friendly alternatives in public health, food production systems, and the bioremediation of contaminated environments, particularly in developing countries [76].

Phages support UN SDG 17 “Partnerships for the Goals” by promoting knowledge sharing and cooperation in global research and technology transfer. This promotes equitable access to phage-based solutions to achieve many of the Sustainable Development Goals in health, agriculture, and environmental sustainability [50].

## 3. Conclusions

In the context of the bactericidal activity of phages, a number of synergies can be identified that may help achieve multiple sustainability goals. Bacteriophage-based biotechnological innovations are crucial for attaining most of the UN SDGs and have the potential to facilitate a rapid transition to a sustainable global economy. However, despite their beneficial impacts on the UN SDGs, it is surprising that research and biotechnological development related to bacteriophages are not included in government policies [49,50]. There are still several challenges in implementing phage uses and applications across various sectors, such as medicine, aquaculture, agriculture, livestock, and bioremediation. These challenges often originate from the lack of clear regulations for each phage administration and/or treatments, which are necessary to ensure their efficacy and safety [73]. Additionally, funding limitations pose a significant barrier to the widespread adoption of phage-based technologies. To successfully implement these innovations, financial and regulatory policies must be created that acknowledge the unique potential of bacteriophages.

Research has shown that phage-based technologies could help combat antimicrobial resistance, enhance global food security in an environmentally sustainable way, and contribute to achieving most of the UN SDGs outlined in the 2030 Agenda. However, raising public awareness about the benefits and safety of phages for global health and sustainability is essential for their widespread acceptance and use in the society.

Phages have been identified as a potentially effective solution to the global threat posed by multidrug-resistant bacteria. Taking into consideration the mounting challenges created by antimicrobial resistance, there is an urgent need to explore phage-based interventions as a means to enhance global health security and to facilitate the development of a safer and healthier future.

## References

[1] Clokie M.R., Millard A.D., Letarov A.V., Heaphy S. (2011). Phages in Nature. Bacteriophage.

[2] Naureen Z., Dautaj A., Anpilogov K., Camilleri G., Dhuli K., Tanzi B., Maltese P.E., Cristofoli F., DeAntoni L., Beccari T. (2020). Bacteriophages Presence in Nature and Their Role in the Natural Selection of Bacterial Populations. Acta Biomed..

[3] Abril A.G., Carrera M., Notario V., Sánchez-Pérez Á., Villa T.G. (2022). The Use of Bacteriophages in Biotechnology and Recent Insights Into Proteomics. Antibiotics.

[4] Jo S.J., Kwon J., Kim S.G., Lee S.J. (2023). The Biotechnological Application of Bacteriophages: What to Do and Where to Go in the Middle of the Post-Antibiotic Era. Microorganisms.

[5] García P., Tabla R., Anany H., Bastias R., Brøndsted L., Casado S., Cifuentes P., Deaton J., Denes T.G., Islam M.A. (2023). ECOPHAGE: Combating Antimicrobial Resistance Using Bacteriophages for Eco-Sustainable Agriculture and Food Systems. Viruses.

[6] Álvarez B., Biosca E.G. (2024). Potential of the Bacteriophage-Based Therapy for a More Eco-Sustainable Agriculture. Int. J. Biol. Nat. Sci..

[7] Haq I.U., Rahim K., Paker N.P. (2024). Exploring the Historical Roots, Advantages and Efficacy of Phage Therapy in Plant Diseases Management. Plant Sci..

[8] United Nations Transforming Our World: The 2030 Agenda for Sustainable Development. A/RES/70/1.2015. https://www.refworld.org/legal/resolution/unga/2015/en/111816.

[9] European Commission (2017). A European One Health Action Plan Against Antimicrobial Resistance (AMR). https://health.ec.europa.eu/system/files/2020-01/amr_2017_action-plan_0.pdf.

[10] Erez Z., Steinberger-Levy I., Shamir M., Doron S., Stokar-Avihail A., Peleg Y., Melamed S., Leavitt A., Savidor A., Albeck S. (2017). Communication Between Viruses Guides Lysis-Lysogeny Decisions. Nature.

[11] Makky S., Dawoud A., Safwat A., Abdelsattar A.S., Rezk N., El-Shibiny A. (2021). The Bacteriophage Decides Own Tracks: When They Are With or Against the Bacteria. Curr. Res. Microb. Sci..

[12] Principi N., Silvestri E., Esposito S. (2019). Advantages and Limitations of Bacteriophages for the Treatment of Bacterial Infections. Front. Pharmacol..

[13] Lin D.M., Koskella B., Lin H.C. (2017). Phage Therapy: An Alternative to Antibiotics in the Age of Multi-Drug Resistance. World J. Gastrointest. Pharmacol. Ther..

[14] Behera M., De S., Ghorai S.M. (2024). The Synergistic and Chimeric Mechanism of Bacteriophage Endolysins: Opportunities for Application in Biotherapeutics, Food, and Health Sectors. Probiotics Antimicrob. Proteins.

[15] Liu S., Lu H., Zhang S., Shi Y., Chen Q. (2022). Phages Against Pathogenic Bacterial Biofilms and Biofilm-Based Infections: A Review. Pharmaceutics.

[16] Biosca E.G., Català-Senent J.F., Figàs-Segura À., Bertolini E., López M.M., Álvarez B. (2021). Genomic Analysis of the First European Bacteriophages With Depolymerase Activity and Biocontrol Efficacy Against the Phytopathogen *Ralstonia solanacearum*. Viruses.

[17] Islam M.M., Mahbub N.U., Shin W.S., Oh M.H. (2024). Phage-Encoded Depolymerases as a Strategy for Combating MultiDrug-Resistant *Acinetobacter baumannii*. Front. Cell Infect. Microbiol..

[18] Zhao J., Wang J., Zhang C., Xu S., Ren H., Zou L., Ma J., Liu W. (2024). Characterization of a *Salmonella abortus equi* phage 4FS1 and Its Depolymerase. Front. Vet. Sci..

[19] Bertolini E., Figàs-Segura À., Álvarez B., Biosca E.G. (2023). Development of TaqMan Real-Time PCR Protocols for Simultaneous Detection and Quantification of the Bacterial Pathogen *Ralstonia solanacearum* and Their Specific Lytic Bacteriophages. Viruses.

[20] Ranveer S.A., Dasriya V., Ahmad M.F., Dhillon H.S., Samtiya M., Shama E., Anand T., Dhewa T., Chaudhary V., Chaudhary P. (2024). Positive and Negative Aspects of Bacteriophages and Their Immense Role in the Food Chain. NPJ Sci. Food.

[21] Rogovski P., Cadamuro R.D., da Silva R., de Souza E.B., Bonatto C., Viancelli A., Michelon W., Elmahdy E.M., Treichel H., Rodríguez-Lázaro D. (2021). Uses of Bacteriophages as Bacterial Control Tools and Environmental Safety Indicators. Front. Microbiol..

[22] Duan Y., Llorente C., Lang S., Brandl K., Chu H., Jiang L., White R.C., Clarke T.H., Nguyen K., Torralba M. (2019). Bacteriophage Targeting of Gut Bacterium Attenuates Alcoholic Liver Disease. Nature.

[23] Grubb D.S., Wrigley S.D., Freedman K.E., Wei Y., Vázquez A.R., Trotter R.E., Wallace T.C., Johnson S.A., Weir T.L. (2020). PHAGE-2 Study: Supplemental Bacteriophages Extend *Bifidobacterium animalis* subsp. *lactis* BL04 Benefits on Gut Health and Microbiota in Healthy Adults. Nutrients.

[24] Zhang Y., Sharma S., Tom L., Liao Y.T., Wu V.C.H. (2023). Gut Phageome—An Insight Into the Role and Impact of Gut Microbiome and Their Correlation With Mammal Health and Diseases. Microorganisms.

[25] Milijasevic M., Veskovic-Moracanin S., Babic Milijasevic J., Petrovic J., Nastasijevic I. (2024). Antimicrobial Resistance in Aquaculture: Risk Mitigation Within the One Health Context. Foods.

[26] Lobo R.R., Faciola A.P. (2021). Ruminalphages—A Review. Front. Microbiol..

[27] Aydin S., Can K., Çalışkan M., Balcazar J.L. (2022). Bacteriophage Cocktail as a Promising Bio-Enhancer for Methanogenic Activities in Anaerobic Membrane Bioreactors. Sci. Total Environ..

[28] Lu S.Y., Liu S., Patel M.H., Glenzinski K.M., Skory C.D. (2023). *Saccharomyces cerevisiae* Surface Display of Endolysin LysKB317 for Control of Bacterial Contamination in Corn Ethanol Fermentations. Front. Bioeng. Biotechnol..

[29] Loc-Carrillo C., Abedon S.T. (2011). Pros and Cons of Phage Therapy. Bacteriophage.

[30] Gundersen M.S., Fiedler A.W., Bakke I., Vadstein O. (2023). The Impact of Phage Treatment on Bacterial Community Structure is Minor Compared to Antibiotics. Sci. Rep..

[31] Koskella B., Hernandez C.A., Wheatley R.M. (2022). Under Standing the Impacts of Bacteriophage Viruses: From Laboratory Evolution to Natural Ecosystems. Annu. Rev. Virol..

[32] Tokuda M., Shintani M. (2024). Microbial Evolution Through Horizontal Gene Transfer by Mobile Genetic Elements. Microb. Biotechnol..

[33] Durbas I., Machnik G. (2022). Phage Therapy: An Old Concept With New Perspectives. J. Appl. Pharm. Sci..

[34] Chen B., Ponce Benavente L., Chittò M., Post V., Constant C., Zeiter S., Nylund P., D’Este M., González Moreno M., Trampuz A. (2024). Combination of Bacteriophages and Vancomycin in a Co-Delivery Hydrogel for Localized Treatment of Fracture-Related Infections. NPJ Biofilms Microbiomes.

[35] Mirzaei A., Esfahani B.N., Ghanadian M., Wagemans J., Lavigne R., Moghim S. (2024). *Alhagi maurorum* Extract in Combination With Lytic Phage Cocktails: A Promising Therapeutic Approach Against Biofilms of Multi-Drug Resistant *P. mirabilis*. Front. Pharmacol..

[36] Rastegar S., Skurnik M., Tadjrobehkar O., Samareh A., Samare-Najaf M., Lotfian Z., Khajedadian M., Hosseini-Nave H., Sabouri S. (2024). Synergistic Effects of Bacteriophage Cocktail and Antibiotics Combinations Against Extensively Drug-Resistant *Acinetobacter baumannii*. BMC Infect. Dis..

[37] Shymialevich D., Wójcicki M., Sokołowska B. (2024). The Novel Concept of Synergically Combining: High Hydrostatic Pressure and Lytic Bacteriophages to Eliminate Vegetative and Spore-Forming Bacteria in Food Products. Foods.

[38] Álvarez B., Biosca E.G. (2017). Bacteriophage-Based Bacterial Wilt Biocontrol for an Environmentally Sustainable Agriculture. Front. Plant Sci..

[39] Álvarez B., Gadea-Pallás L., Rodríguez A., Vicedo B., Figàs-Segura À., Biosca E.G. (2022). Viability, Stability and Biocontrol Activity *In Planta* of Specific *Ralstonia solanacearum* Bacteriophages After Their Conservation Prior to Commercialization and Use. Viruses.

[40] Jo S.J., Giri S.S., Lee S.B., Jung W.J., Park J.H., Hwang M.H., Park D.S., Park E., Kim S.W., Jun J.W. (2024). Optimization of the Large-Scale Production for *Erwinia amylovora* Bacteriophages. Microb. Cell Fact..

[41] Krysiak-Baltyn K., Martin G.J.O., Gras S.L. (2018). Computational Modelling of Large Scale Phage Production Using a Two-Stage Batch Process. Pharmaceuticals.

[42] Álvarez B., López M.M., Biosca E.G. (2019). Biocontrol of the Major Plant Pathogen *Ralstonia solanacearum* in Irrigation Water and Host Plants by Novel Water Borne Lytic Bacteriophages. Front. Microbiol..

[43] Oechslin F. (2018). Resistance Development to Bacteriophages Occurring During Bacteriophage Therapy. Viruses.

[44] Lopatina A., Tal N., Sorek R. (2020). Abortive Infection: Bacterial Suicide as an Antiviral Immune Strategy. Annu. Rev. Virol..

[45] Zou H., Huang X., Xiao W., He H., Liu S., Zeng H. (2025). Recent Advancements in Bacterial Anti-Phage Strategies and the Underlying Mechanisms Altering Susceptibility to Antibiotics. Microbiol. Res..

[46] Chung K.M., Nang S.C., Tang S.S. (2023). The Safety of Bacteriophages in Treatment of Diseases Caused by MultiDrug-Resistant Bacteria. Pharmaceuticals.

[47] Liu D., van Belleghem J.D., de Vries C.R., Burgener E., Chen Q., Manasherob R., Aronson J.R., Amanatullah D.F., Tamma P.D., Suh G.A. (2021). The Safety and Toxicity of Phage Therapy: A Review of Animal and Clinical Studies. Viruses.

[48] Faltus T. (2024). The Medicinal Phage-Regulatory Roadmap for Phage Therapy Under EU Pharmaceutical Legislation. Viruses Mar..

[49] Yang Q., Le S., Zhu T., Wu N. (2023). Regulations of Phage Therapy Across the World. Front. Microbiol..

[50] Crowther T.W., Rappuoli R., Corinaldesi C., Danovaro R., Donohue T.J., Huisman J., Stein L.Y., Timmis J.K., Timmis K., Anderson M.Z. (2024). Scientists’Call to Action: Microbes, Planetary Health, and the Sustainable Development Goals. Cell.

[51] Mohsin S., Amin M.N. (2023). Superbugs: A Constraint to Achieving the Sustainable Development Goals. Bull. Natl. Res. Cent..

[52] Samson R., Dharne M., Khairnar K. (2024). Bacteriophages: Status Quo and Emerging Trends Toward One Health Approach. Sci. Total Environ..

[53] Summer E.J., Liu M. (2016). Application of Bacteriophages for the Control of Unwanted Bacteria in Biofuel Production Mediated by Non-Bacterial Reactive Agents. U.S. Patent.

[54] González Biosca E., López González M.M., Álvarez Ortega B. (2017). Procedimiento para la Prevención y/o el Control Biológico de la Marchitez Causada por Ralstonia solanacearum, a Través del Uso de Bacteriófagos Útiles para Ello y Composiciones de los Mismos. Spain Patent.

[55] González Biosca E., López González M.M., Álvarez Ortega B. (2019). Method for the Prevention and/or the Biological Control of Bacterial Wilt Caused by Ralstonia solanacearum, via the Use of Bacteriophages Suitable for This Purpose and Compositions Thereof. U.S. Patent.

[56] González Biosca E., López González M.M., Álvarez Ortega B. (2020). Method for the Prevention and/or the Biological Control of Bacterial Wilt Caused by Ralstonia solanacearum, via the Use of Bacteriophages Suitable for this Purpose and Compositions Thereof. Eur. Patent.

[57] Gutiérrez D., Fernández L., Rodríguez A., García P. (2019). Role of Bacteriophages in the Implementation of a Sustainable Dairy Chain. Front. Microbiol..

[58] Siyanbola K.F., Ejiohuo O., Ade-adekunle O.A., Adekunle F.O., Onyeaka H., Furr C.-L.L., Hodges F.E., Carvalho P., Oladipo E.K. (2024). Bacteriophages: Sustainable and Effective Solution for Climate-Resilient Agriculture. Sustain. Microbiol..

[59] Vikram A., Callahan M.T., Woolston J.W., Sharma M., Sulakvelidze A. (2022). Phage Biocontrol for Reducing Bacterial Food Borne Pathogens in Produce and other Foods. Curr. Opin. Biotechnol..

[60] Batinovic S., Wassef F., Knowler S.A., Rice D.T.F., Stanton C.R., Rose J., Tucci J., Nittami T., Vinh A., Drummond G.R. (2019). Bacteriophages in Natural and Artificial Environments. Pathogens.

[61] Holtappels D., Fortuna K., Lavigne R., Wagemans J. (2021). The Future of Phage Biocontrol in Integrated Plant Protection for Sustainable Crop Production. Curr. Opin. Biotechnol..

[62] Garvey M. (2022). Bacteriophages and Food Production: Biocontrol and Bio-Preservation Options for Food Safety. Antibiotics.

[63] Biosca E.G., Delgado-Santander R., Morán F., Figàs-Segura À., Vázquez R., Català-Senent J.F., Álvarez B. (2024). First European *Erwinia amylovora* Lytic Bacteriophage Cocktails Effective in the Host: Characterization and Prospects for Fire Blight Biocontrol. Biology.

[64] Suja E., Gummadi S.N. (2024). Advances in the Applications of Bacteriophages and Phage Products Against Food-Contaminating Bacteria. Crit. Rev. Microbiol..

[65] Pirnay J.P., Djebara S., Steurs G., Griselain J., Cochez C., DeSoir S., Glonti T., Spiessens A., Vanden Berghe E., Green S. (2024). Personalized Bacteriophage Therapy Outcomes for 100 Consecutive Cases: A Multicentre, Multinational, Retrospective Observational Study. Nat. Microbiol..

[66] McCammon S., Makarovs K., Banducci S., Gold V. (2023). Phage Therapy and the Public: Increasing Awareness Essential to Widespread Use. PLoS ONE.

[67] Thompson T., Kilders V., Widmar N., Ebner P. (2024). Consumer Acceptance of Bacteriophage Technology for Microbial Control. Sci. Rep..

[68] Heller D.M., Sivanathan V., Asai D.J., Hatfull G.F. (2024). SEA-PHAGES and SEA-GENES: Advancing Virology and Science Education. Annu. Rev. Virol..

[69] Maicas S., Fouz B., Figàs-Segura À., Zueco J., Rico H., Navarro A., Carbó E., Segura-García J., Biosca E.G. (2020). Implementation of Antibiotic Discovery by Student Crowdsourcing in the Valencian Community Through a Service Learning Strategy. Front. Microbiol..

[70] Timmis K., Hallsworth J.E., McGenity T.J., Armstrong R., Colom M.F., Karahan Z.C., Chavarría M., Bernal P., Boyd E.S., Ramos J.L. (2024). A Concept for International Societally Relevant Microbiology Education and Microbiology Knowledge Promulgation in Society. Microb. Biotechnol..

[71] Hatfull G.F. (2021). Wildy Prize Lecture, 2020-2021: Who Wouldn’t Want to Discover a New Virus?. Microbiology.

[72] Citizen Phage Library (2025). Developing Therapeutic Phages to Fight Antimicrobial Resistance With Citizen Science. https://www.citizenphage.com.

[73] Hitchcock N.M., Devequi Gomes Nunes D., Shiach J., Valeria Saraiva Hodel K., Dantas Viana Barbosa J., Alencar Pereira Rodrigues L., Coler B.S., Botelho Pereira Soares M., Badaró R. (2023). Current Clinical Landscape and Global Potential of Bacteriophage Therapy. Viruses.

[74] Khan A.N., Soomro M.A., Khan N.A., Bodla A.A. (2024). Psychological Dynamics of over Qualification: Career Anxiety and Decision Commitment in STEM. BMC Psychol..

[75] Sieiro C., Areal-Hermida L., Pichardo-Gallardo Á., Almuiña-González R., de Miguel T., Sánchez S., Sánchez-Pérez Á., Villa T.G. (2020). A Hundred Years of Bacteriophages: Can Phages Replace Antibiotics in Agriculture and Aquaculture?. Antibiotics.

[76] Khalid A., Lin R.C.Y., Iredell J.R. (2021). A Phage Therapy Guide for Clinicians and Basic Scientists: Background and Highlighting Applications for Developing Countries. Front. Microbiol..

[77] Huang D., Xia R., Chen C., Liao J., Chen L., Wang D., Alvarez P.J.J., Yu P. (2024). Adaptive Strategies and Ecological Roles of Phages in Habitats Under Physicochemical Stress. Trends Microbiol..

[78] Fiedler A.W., Gundersen M.S., Vo T.P., Almaas E., Vadstein O., Bakke I. (2023). Phage Therapy Minimally Affects the Water Microbiota in an Atlantic Salmon (*Salmo salar*) Rearing System While Still Preventing Infection. Sci. Rep..

